# Added value of lymphocyte subpopulations in the classification of Sjögren's syndrome

**DOI:** 10.1038/s41598-023-31782-7

**Published:** 2023-04-27

**Authors:** Filipe Barcelos, Carlos Brás-Geraldes, Catarina Martins, Ana-Luísa Papoila, Ricardo Monteiro, Joana Cardigos, Nathalie Madeira, Nuno Alves, José Vaz-Patto, Jaime Cunha-Branco, Luís-Miguel Borrego

**Affiliations:** 1 Comprehensive Health Research Centre, CEDOC, Chronic Diseases Research Center, Immunology, NOVA Medical School, Universidade Nova de Lisboa, Campo Dos Mártires da Pátria, 130, 1169-056 Lisbon, Portugal; 2 Comprehensive Health Research Centre (CHRC), NOVA Medical School, FCM, Universidade Nova de Lisboa, Lisbon, Portugal; 3 Rheumatology Department, Instituto Português de Reumatologia, Lisbon, Portugal; 4 Rheumatology Department, Hospital CUF Descobertas, Lisbon, Portugal; 5 ISEL-Instituto Superior de Engenharia de Lisboa, Instituto Politécnico de Lisboa, Lisbon, Portugal; 6 Centro de Estatística E Aplicações, CEAUL, Universidade de Lisboa, Lisbon, Portugal; 7 NOVA Medical School, FCM, Universidade Nova de Lisboa, Lisbon, Portugal; 8 Ophthalmology Department, Centro Hospitalar de Lisboa Central, Hospital de Santo António Dos Capuchos, Lisbon, Portugal; 9 Ophthalmology Department, Hospital CUF Descobertas, Lisbon, Portugal; 10 Chronic Diseases Research Center, NOVA Medical School, FCM, Universidade Nova de Lisboa, Lisbon, Portugal; 11 Rheumatology Department, Centro Hospitalar de Lisboa Ocidental, Hospital de Egas Moniz, Lisbon, Portugal; 12 Immunoalergy Department, Hospital da Luz Lisboa, Lisbon, Portugal

**Keywords:** SjÃ¶gren's disease, Rheumatic diseases

## Abstract

Sjögren's Syndrome (SjS) is a chronic systemic immune-mediated inflammatory disease characterized by lymphocytic infiltration and consequent lesion of exocrine glands. SjS diagnosis and classification remains a challenge, especially at SjS onset, when patients may have milder phenotypes of the disease or uncommon presentations. New biomarkers are needed for the classification of SjS, thus, we aimed to evaluate the added-value of lymphocyte subpopulations in discriminating SjS and non-Sjögren Sicca patients. Lymphocyte subsets from 62 SjS and 63 Sicca patients were characterized by flow cytometry. The 2002 AECG and the 2016 ACR/EULAR SjS classification criteria were compared with clinical diagnosis. The added discriminative ability of joining lymphocytic populations to classification criteria was assessed by the area under the Receiver-Operating-Characteristic Curve (AUC). Considering clinical diagnosis as the gold-standard, we obtained an AUC = 0.952 (95% CI: 0.916–0.989) for AECG and an AUC = 0.921 (95% CI: 0.875–0.966) for ACR/EULAR criteria. Adding Tfh and Bm1 subsets to AECG criteria, performance increased, attaining an AUC = 0.985 (95% CI: 0.968–1.000) (p = 0.021). Th1/Breg-like CD24^hi^CD27^+^ and switched-memory B-cells maximized the AUC of ACR/EULAR criteria to 0.953 (95% CI: 0.916–0.990) (p = 0.043). Our exploratory study supports the potential use of lymphocyte subpopulations, such as unswitched memory B cells, to improve the performance of classification criteria, since their discriminative ability increases when specific subsets are added to the criteria.

## Introduction

Sjögren's Syndrome (SjS) is a chronic systemic immune-mediated inflammatory disease characterized by lymphocytic infiltration and consequent lesion of exocrine glands^1^. The most common symptoms are xerostomia and xerophthalmia but extra-glandular symptoms occur in up to 50% of patients^2^.

T-cells infiltrate target-organs, such as the salivary and lachrymal glands, and these follicular T-cells (Tfh) are a major source of IL-21, a cytokine that mediates B-cell differentiation and proliferation and promotes the ectopic formation of germinal centre (GC)-like structures^3^. In the GC-like structures, Tfh-B-cell interactions result in amplification of B-cell activation^4^. B-cells hyperactivity is the hallmark of SjS, playing a major role in pathogenesis and clinical evolution^5^. Tregs, who play a crucial role in maintaining self-tolerance and controlling the expansion and activation of autoreactive CD4^+^ effector T-cells and other immune cells^6^, are thought to be impaired in SjS^7^.

In addition to symptoms of dryness, objective signs are sought to confirm salivary or lachrymal gland dysfunction^8,9^. For dry eye, these include evidence of keratoconjunctivitis *sicca* (quantified as a score, such as the Ocular Staining Score^10^) and Schirmer's test^9^. Decreased salivary glands’ (SG) function may be assessed by sialometry or indirectly suspected through sonographic changes of the SG^11^.

The infiltration and proliferation of autoreactive B and T-cells in the exocrine glands of SjS patients is reflected in the main markers of SjS—anti-SSA and anti-SSB antibodies, present in 50–70% and 33–50% of patients, respectively^12^, and by the presence of focal lymphocytic infiltration in the minor labial SG biopsy^13^.

## Theory and calculation

Classification criteria are useful in clinical practice as a guidance to support diagnosis. The 2002 American-European Consensus Group (AECG) SjS classification criteria^14^ have been widely used since their presentation, with over 1500 references^15^. The new classification criteria proposed in 2016 by the American College of Rheumatology/European League Against Rheumatism (ACR/EULAR)^16^ were intended to replace the AECG criteria (Supplementary Table 1). Both criteria include the most specific finding for SjS diagnosis, which is the demonstration of focal lymphocytic infiltration on histology of minor labial SG biopsy^13^. The main serological markers of SjS, anti-SSA, present in 50–70% and 33–50% of patients, respectively^12^, have also been included in both criteria.

Nevertheless, despite the high sensitivity and specificity of both criteria sets, not all patients with a clinical diagnosis of SjS will fulfil classification criteria^17^. Efforts to increase the performance of SjS classification criteria have been made, such as the exploration of the effect of the inclusion of salivary gland sonography features in the 2016 ACR/EULAR classification criteria^18^.

Changes in the distribution of peripheral blood B-cell subsets are typical of SjS, with increased naïve B-cells and decreased memory B-cells, particularly unswitched memory^19^. Binard studied the effect of adding a naive-to-mature B-cells’ ratio to the 2002 AECG classification criteria, reporting an increase in their performance for the diagnosis^20^. However, in a later study, these parameters performed poorly in a distinct clinical setting^21^.

T-cells are crucial in SjS pathogenesis^4^, and a recent study that assessed the utility of lymphocyte phenotype profile to differentiate SjS from sicca syndrome focused on these cells^22^. However, to our knowledge, no study has been made regarding their use as a biomarker in SjS classification criteria.

We aimed to evaluate the added value, regarding the discriminative ability to differentiate between SjS and non-Sjögren sicca patients (designated as “Sicca” in this work), resulting from including lymphocytes subpopulations in the ACR/EULAR and AECG criteria. For that purpose, a comparison of B and T-cells subsets’ distribution between SjS and Sicca patients was also performed.

## Results

### Participants

Sixty-two patients were included in the SjS group and 63 in the Sicca group.

Clinical and demographic data of SjS and Sicca are presented in Table 1.

**Table 1:** SjS and Sicca Patients' characteristics.

	SjS	Sicca
	n = 62	n = 63
Age, mean (SD) (years)	58.1 (12.1)	60.9 (10.3)
Symptom duration, mean (SD) (years)	11.7 (7.6)	9.7 (4.9)
Ocular symptoms, n (%)	59 (95.2)	60 (95.2)
Oral symptoms, n (%)	60 (96.8)	60 (95.2)
Low Schirmer's, n (%)	34 (55.7)^a^	29 (46.8)^b^
Positive Corneal Staining, n (%)	20 (32.8)^a^	11 (17.7)^b^
Low Unstimulated Salivary Flow, n (%)	41 (66.1)	38 (30.3)
Focus score ≥ 1, n (%)	43 (71.7) ^c^	0 (0)
Anti-SSA, n (%)	37 (67.3)	1 (1.6)
Anti-SSB, n (%)	18 (32.7) ^d^	1 (1.8) ^e^

^a^n = 61. ^b^n = 62. ^c^n = 60. ^d^n = 55. ^e^n = 56.

SjS, Sjogren’s syndrome; SSA/SSB, Sjögren’s syndrome antigen A and B.

### Lymphocyte subsets

Complete results are presented in Table 2 and Supplementary Table 2.

**Table 2:** Comparison of T and B-cell subsets percentages in SjS and Sicca groups.

Lymphocyte subsets or combinations Cells %, median (25–75th percentile)	SjS n = 62	Sicca n = 63	SjS vs Sicca *p* values
Lymphocytes	30.6 (11.0)*	31.4 (8.7)*	0.745
T-cells	75.0 (68.2, 78.0)	72.2 (67.6, 77.0)	0.185
CD4^+^	61.2 (53.7, 68.1)	65.9 (60.3, 70.0)	**0.009**
CD8^+^	38.9 (11.4)*	34.0 (9.1)*	**0.012**
B-cells	10.2 (7.5, 14.1)	11.2 (8.5, 14.6)	0.411
T-cells' subpopulations			
Th1	38.7 (29.7, 45.6)	40.4 (30.2, 47.4)	0.555
Th17	19.8 (14.2, 28.4)	22.3 (16.6, 28.6)	0.293
Treg	8.4 (6.6, 10.0)	7.6 (6.9, 8.7)	**0.059**
Tfh (CXCR5^+^CD4^+^)	19.4 (5.8)*	18.4 (4.7)*	0.397
Tfh1	35.4 (7.8)*	32.3 (6.9)*	**0.025**
Tfh17	21.9 (6.6)*	23.2 (5.6)*	0.165
Tfc (CXCR5^+^CD8^+^)	2.6 (1.9, 4.2)	2.63 (1.88, 4.07)	0.865
B-cell's subpopulations			
IgD/CD27			
Naive	68.5 (52.5, 77.7)	66.6 (48.6, 75.1)	0.369
Memory	29.3 (20.0, 44.1)	31.4 (23.3, 45.2)	0.239
Unswitched	13.6 (8.9, 21.6)	17.5 (11.2, 24.5)	**0.084**
Switched	14.4 (9.7, 21.9)	14.7 (10.9, 21.6)	0.724
Bm1–5			
Bm1	10.0 (5.6, 14.9)	13.9 (8.1, 19.5)	**0.003**
Bm2	61.4 (49.9, 67.2)	59.8 (47.5, 66.5)	0.673
Bm2'	8.5 (5.9) *	5.9 (3.3) *	**0.046**
Bm3 + Bm4	1.34 (0.93, 3.28)	1.36 (0.87, 1.85)	0.348
eBm5	8.5 (6.1, 12.5)	8.6 (6.3, 12.6)	0.841
Bm5	6.9 (4.4, 12.4)	7.8 (5.1, 10.6)	0.927
Bm2 + Bm2'	71.2 (56.2, 79.2)	67.1 (52.0, 73.4)	0.146
eBm5 + Bm5	16.8 (10.8, 24.9)	16.7 (12.4, 25.2)	0.884
Bm2 + Bm2'/Bm5 + eBm5	3.9 (2.2, 7.2) *	4.1 (2.0, 5.9) *	0.641
CD24^hi^CD38^hi^	5.6 (2.2, 8.5) *	4.1 (2.7, 5.3) *	0.061
CD24^hi^CD27^+^	16.9 (12.2, 25.4)	21.3 (14.3, 35.4)	**0.024**
T-cell/B-cell ratios			
T_h_1/CD24^hi^CD27^+^	2.23 (1.22, 3.60)	1.60 (1.09, 2.70)	**0.033**
T_h_17/Tregs	0.26 (0.18, 0.40)	0.35 (0.22, 0.44)	**0.021**

Bold numbers represent significance (p-values < 0.05) or relevance (p-values < 0.100); SjS, Sjogren’s syndrome.

*indicates Mean and Standard Deviation.

#### CD4^+^ and CD8^+^ T-cell subsets

SjS patients presented lower CD4 + T-cells' percentages and absolute counts compared to Sicca (p = 0.009 and p = 0.051), and higher CD8 + T-cells percentages (p = 0.012).

Tregs counts were similar in both patients' groups (p = 0.436), although SjS patients presented evidence, although weak, of higher percentages (p = 0.059), compared to Sicca.

Tfh and Tfc cells were similar in both groups of patients. Concerning Tfh subsets, Tfh1 percentages were higher in SjS than in Sicca (p = 0.025), and Tfh17 absolute counts were decreased in SjS (p = 0.05) (Table 2, Supp Table 2).

#### B-cell subsets according to IgD and CD27 classification

SjS patients had lower absolute counts of B-cells in comparison to Sicca patients (p = 0.152), and a significant decrease was found in the absolute counts of CD19^+^CD27^+^ memory B-cells' in SjS (50 vs 66 cells/µl, p = 0.021), more pronounced in the CD19^+^IgD^+^CD27^+^ unswitched memory B-cells subset (23 vs 35 cells/µl, p = 0.009) (Table 2 and Supp 2). CD19^+^IgD^+^CD27^+^ unswitched memory B-cells' percentages were also lower in SjS patients, with a trend to significance compared to Sicca (13.6% vs 17.5%, p = 0.084). Regarding naïve B-cells, there were no significant differences between SjS and Sicca patients (Table 2, Supplementary Table 2).

#### Bm1-Bm5 classification of mature B cells

The percentages and absolute counts of Bm1 cells were lower in SjS patients than in Sicca (p = 0.003 and p < 0.001, respectively) (Table 2, Supplementary Table 2).

Regarding Bm2, no significant differences were found between groups. SjS patients presented higher Bm2´cells' percentages compared to Sicca (p = 0.046), without differences in counts.

SjS patients presented lower absolute counts of eBm5 and Bm5 cells compared to Sicca, with a trend to significance (p = 0.089 and p = 0.079, respectively), whereas percentages were similar.

No differences were found in the Bm2 + Bm2'/Bm5 + eBm5 ratio between SjS and Sicca patients.

#### Other B-cell subsets

When analysing subsets related to regulatory B-cells, CD24^hi^CD27^+^ B-cells were decreased in SjS compared to Sicca (p = 0.001 for absolute counts and p = 0.024 for percentages).

CD24^hi^CD38^hi^ B-cells absolute counts were similar in both groups, and weak evidence of higher percentages was identified in SjS patients compared to Sicca patients (5.6% vs 4.1%, p = 0.061).

### Performance of T and B-cells' subsets in the discrimination between SjS and Sicca patients

Lymphocyte subsets were compared between SjS and Sicca, and AUC's were estimated considering the expert clinical diagnosis as the gold standard (Table 3).

**Table 3:** Area Under the Curve (AUC) receiver operating characteristic curve (ROC) of the comparison of T and B-cell subsets percentages in SjS (clinical diagnosis) and Sicca groups (GS vs Sicca). Added-value of the addition of each cell subset to the AECG (AUC and p-value) and the ACR/EULAR criteria (AUC and p-value).

Lymphocyte subsets or combinations AUC, 95%CI	GS vs Sicca AUC	Added to 2002 AECG		Added to 2016 ACR/EULAR	
		AUC	*p* values	AUC	*p* values
Lymphocytes					
T-cells	0.57 (0.47—0.67)	0.958(0.919—0.998)	0.625	0.932 (0.884—0.98)	0.440
B-cells	0.54 (0.44—0.65)	0.985(0.968—1)	**0.0227**	0.944 (0.900—0.988)	0.163
T-cell subpopulations					
Th1	0.53 (0.43—0.63)	0.959 (0.921—0.996)	0.542	0.931 (0.882—0.979)	0.496
Th17	0.56 (0.45—0.66)	0.950 (0.904—0.996)	0.825	0.922 (0.867—0.978)	0.915
Treg	0.60 (0.50—0.70)	0.978 (0.951—1)	**0.081**	0.932 (0.877—0.987)	0.580
Tfh (CXCR5^+^)	0.54 (0.44—0.65)	0.977 (0.953—1)	**0.053**	0.944 (0.898—0.989)	0.190
Tfh1	0.62 (0.52—0.72)	0.956 (0.919—0.993)	0.640	0.924 (0.872—0.976)	0.824
Tfh17	0.57 (0.47—0.67)	0.957 (0.916—0.999)	0.714	0.925 (0.870—0.980)	0.804
Tfc (CXCR5^+^CD8^+^)	0.51 (0.41—0.61)	0.954 (0.914—0.995)	0.860	0.922 (0.868—0.975)	0.945
B-cells' subpopulations					
IgD/CD27					
Naive	0.55 (0.45—0.65)	0.964 (0.928—1)	0.352	0.938 (0.892—0.984)	0.272
Memory	0.56 (0.46—0.66)	0.965 (0.930—1)	0.307	0.939 (0.894—0.984)	0.232
Unswitched	0.59 (0.49—0.69)	0.968 (0.936—1)	0.206	0.933 (0.885—0.981)	0.404
Switched	0.52 (0.42—0.62)	0.960 (0.920 – 1)	0.640	0.939 (0.892—0.986)	0.269
Bm1–5					
Bm1	0.66 (0.56—0.75)	0.975 (0.95—1)	**0.062**	0.943 (0.903—0.982)	**0.087**
Bm2	0.52 (0.42—0.62)	0.967 (0.935—1)	0.207	0.948 (0.908—0.987)	**0.077**
Bm2'	0.60 (0.50—0.71)	0.963 (0.925—1)	0.422	0.935 (0.887—0.983)	0.362
Bm3 + Bm4	0.45 (0.35—0.55)	0.966 (0.934—0.997)	0.199	0.945 (0.903—0.987)	0.125
eBm5	0.51 (0.41—0.61)	0.947 (0.900—0.994)	0.668	0.941 (0.896—0.986)	0.215
Bm5	0.51 (0.40—0.61)	0.957 (0.915—0.999)	0.726		0.477
Bm2 + Bm2'	0.58 (0.47—0.68)	0.965 (0.930—1)	0.307	0.943 (0.900—0.985)	0.139
eBm5 + Bm5	0.49 (0.39—0.60)	0.958 (0.916—0.999)	0.688	0.936 (0.888—0.983)	0.343
Bm2 + Bm2'/Bm5 + eBm5	0.48 (0.37—0.58)	0.960 (0.920—0.999)	0.570	0.939 (0.894—0.985)	0.238
CD24^hi^CD38^hi^	0.60 (0.49—0.70)	0.960 (0.918—1)	0.604	0.939 (0.891—0.987)	0.295
CD24^hi^CD27^+^	0.62 (0.52—0.72)	0.966 (0.932—1)	0.275	0.938 (0.894—0.982)	0.226
T-cells/B-cells ratios					
T_h_1/CD24^hi^CD27^+^	0.61 (0.51—0.71)	0.967 (0.933—1)	0.257	0.939 (0.896—0.982)	0.180
T_h_17/Tregs	0.62 (0.52—0.72)	0.967 (0.934—1)	0.223	0.952 (0.912—0.992)	**0.075**

Bold numbers represent significance (p-values < 0.05) or relevance (p-values < 0.100).

GS, Gold-Standard; AUC, area under the Receiver-Operating-Characteristic Curve; IC: Interval of Confidence, AECG, American-European Consensus Group classification criteria; ACR/EULAR, American College of Rheumatology/European League Against Rheumatism classification criteria.

Considering subsets' percentages, most AUC's obtained were lower than 0.60, with the highest being 0.66 for Bm1 cells, and 0.62 for Tfh1 and CD24^hi^CD27^+^. For absolute counts, higher AUC values were obtained for the Bm1 (0.675) and CD24^hi^CD27^+^(0.668) B-cells (Supplementary Table 3).

Additionally, the Th1/CD24^hi^CD27^+^ B-cells ratio was increased in SjS, compared to Sicca patients (p = 0.033), and for the Th17/Treg ratio, a significantly lower value was also found in SjS patients (p = 0.021).

### Performance of AECG and ACR/EULAR classification criteria compared to expert clinical diagnosis

The AECG and ACR/EULAR criteria were compared with the GS to assess the discriminatory power between patients with SjS and Sicca, resulting in agreement rates of 95.2% and 90.3%, respectively. Both classification criteria agreed in 95.2% of cases. An analysis of the discriminatory ability of the classification criteria was also carried out considering the GS, with an AUC = 0.952 (95%CI: 0.916–0.989) for the AECG criteria and an AUC = 0.921 (95%CI: 0.853–0.954) for the 2016 ACR/EULAR criteria.

### Added value on the discrimination between Sjögren and Sicca patients resulting from the inclusion of lymphocytes subpopulations in both classification criteria

Following a univariable analysis where the expert clinical diagnosis was considered as the dependent variable, some of the lymphocyte subpopulations, namely Tfh, memory B-cells (total and with switch), Bm1, CD24^hi^CD27^+^ B-cells, and the Th1/Breg CD24^hi^CD27^+^ ratio were selected for the multivariable analysis.

Regarding the multivariable model where those lymphocyte subpopulations were added to the 2002 AECG criteria, only the variables Tfh and Bm1 (both in percentages) remained in the final model, and an AUC of 0.985 (95%CI: 0.968–1.000; p = 0.021) was achieved. For the 2016 ACR/EULAR criteria, the variables Th1/Breg CD24^hi^CD27^+^ and absolute counts of switched memory B-cells (dichotomized with a cut-off = 25 cells/µl) maximized the AUC of the multivariable model to 0.953 (95%CI: 0.916–0.990; p = 0.043).

In both cases, the discrimination ability (SjS vs Sicca) of the two multivariable models led to results with statistical significance (p = 0.021 for the AECG, and p = 0.043 for the ACR/EULAR criteria).

## Discussion

SjS diagnosis and classification remains a challenge, especially at the onset of the illness, when patients may have milder phenotypes of the disease or uncommon presentations^23^. In the absence of SjS diagnostic criteria, clinicians often use classification criteria for guidance in making the diagnosis. However, these are not designed to be used for clinical diagnosis or applied to individual patients but intended to identify well-defined homogenous cohorts for clinical research^24^. SjS criteria have been revised with improved methodology, although there is still a margin for improvement^16,25^.

Several authors report the need to improve the value of these criteria, mainly to allow an early diagnosis, because not all patients who have clinical, serological, imaging and/or functional characteristics suggestive of SjS effectively fulfil criteria, and can be erroneously managed^17,26^.

One of our goals was to measure the performance of AECG and ACR/EULAR criteria compared to the gold standard for SjS diagnosis. Here we found an agreement rate of 95.2% of 2002 AECG in discriminating between SjS and Sicca, which was in line with the study of Zaho et al^27^. Concerning the 2016 ACR/EULAR criteria, we found a lower agreement rate, of 92.0%, in discriminating between SjS and Sicca.

Generally, the ACR/EULAR criteria had been reported to be slightly more sensitive than the AECG criteria. A study conducted in Japan that compared the performance of 2016 ACR/EULAR, the 2002 AECG criteria, the 2012 ACR criteria and the Japanese criteria considering physician diagnosis as reference standard concluded that ACR/EULAR criteria were the most sensitive but also the least specific of the criteria^28^. A Korean study^29^ which compared the ACR/EULAR criteria, the 2012 ACR criteria and the AECG criteria also showed that the ACR/EULAR had higher sensitivity and lower specificity compared with both previous criteria sets. Billings^15^ compared the ACR/EULAR and the AECG criteria, which showed similar performance, without evidence of the superiority of the ACR/EULAR set. Le Goff^30^ also found excellent agreement between both criteria sets, with the ACR/EULAR criteria being slightly more sensitive, allowing the classification of some patients with early disease and prominent systemic features.

Several groups have suggested that the performance of the ACR/EULAR criteria could be improved with the inclusion of SG ultrasonography^18,25,30^. Considering the distinctive lymphocyte profile in SjS^31^, we sought to explore the added value of these variables to the ACR/EULAR and AECG SjS classification criteria.

Our SjS cohort had decreased B-cells' counts and presented the distinctive B-cells profile classically described in the disease—lower levels of memory subsets and increased naïve and transitional subsets, as previously reported using the IgD/CD27 classification^31,32^. Regarding the Bm1-Bm5 classification, Bm2 and Bm2' B-cells have been reported to be increased in SjS, whereas Bm5 and eBm5 were decreased^33^, a tendency we have confirmed in our study. We highlight as well a decrease in percentages and absolute counts of Bm1 cells in SjS.

The increase in naïve mature B-cells in SjS has been attributed to the impairment of early B-cell tolerance checkpoints^5^. The mobilization of self-reactive naïve B-cells from the bone marrow to the periphery is increased in SjS^5^, and recently Glauzy^34^ found that among the expanded naïve B-cells in SjS, most clones were polyreactive, pointing to their emergence from defective central and peripheral B-cell tolerance checkpoints in SjS patients. Also, there is increased migration of memory B-cells to the affected SG^35^, accompanied by a shift in B-cell differentiation towards plasma cells^36^.

In our study, CD24^hi^CD27^+^ B-cells, a population of memory B-cells known to be enriched in regulatory cells and the human equivalent of B10 cells^37^, were significantly decreased in SjS patients, although our results are difficult to interpret as functional assays for IL-10-production were not performed. Interest in CD24^hi^CD27^+^ regulatory B cells has grown lately, and this subset has been implicated in other rheumatic diseases, such as Systemic Lupus Erythematosus (SLE)^38^ and Rheumatoid Arthritis (RA)^39,40^. Nevertheless, some data suggest that changes in the "mother population", CD24^hi^CD27^+^ B-cells, may correlate with changes in the IL10^+^CD24^hi^CD27^+^ B-cells, therefore providing a simpler method to address Bregs in a clinical setting^38,40^. In the same line, we have previously reported a negative correlation between CD24^hi^CD27^+^ B-cells and disease activity in anti-SSA-positive SjS patients^41^.

The increased Th1/CD24^hi^CD27^+^ B-cells ratio in our SjS patients may represent an imbalance between Breg-enriched populations and effector Th1 cells.

In SjS patients, we also found T-cell lymphopenia. Lymphopenia in SjS has been attributed to the migration of peripheral blood CD4^+^ T-cells to the exocrine glands^42^ and therefore may traduce a more active disease profile. Increased peripheral Tfh cells have been reported in SjS patients^43^, although in our SjS population only the Tfh1 subset presented increased percentages compared to Sicca, whereas Tfh17 presented lower percentages and absolute counts. Additionally, Tfh1 had one of the highest AUC in discriminating SjS from Sicca. This was not surprising considering the crucial role of Tfh cells in regulating immune responses, allowing B cell differentiation towards memory B cells and plasma cells within secondary lymphoid follicles^44^.

Regarding regulatory T-cells (Tregs), our SjS patients showed a tendency to increased percentages. Tregs’ frequency in SjS is variable in the literature, but SjS patients seem to present Tregs deficiencies^45^, contributing to disease pathogenesis. Therefore, an increased number of Treg cells in SjS does not mean that these cells can suppress the immune response. Additionally, the decreased Th17/Treg ratio found in our SjS population was due to a relative increase in Tregs, as opposed to Th17, whose numbers were in line with the global T-cell lymphopenia.

We also aimed to evaluate the added value on discrimination between SjS and Sicca of the inclusion of lymphocytes subpopulations in both criteria.

It has been previously reported that a high (Bm2 + Bm2′)/(eBm5 + Bm5) ratio is more frequent in SjS both compared to RA, SLE, Sicca patients and healthy subjects, and could constitute a diagnostic tool^20,21^. However, Cornec et al^21^ reported that evaluating this ratio, although valuable for the individual patient, had a small diagnostic weight compared to other items of AECG CC, as adding an item consisting of a (Bm2 + Bm2′)/(eBm5 + Bm5) ratio ≥ 5 did not significantly modify the performance of the criteria.

Mingueneau^42^ recently published the first attempt of a global evaluation, using 34 distinct protein markers characterized by mass cytometry and using bioinformatics analysis to recognize multiple distinct cell subsets. A blood 4-cell disease signature including plasmacytoid dendritic cell, CD4^+^ T cell, memory B cell, and human leukocyte antigen (HLA)-DR^+^CD4^+^ T cells was defined in this manner and was able to diagnose SjS with good accuracy in two different cohorts of patients (AUC 0.86–0.89).

Using conventional cytometry, we have identified several lymphocyte subsets that showed a reasonable accuracy in distinguishing SjS from Sicca patients. When some of these lymphocyte subsets were added to the classification criteria, they were able to increase the performance of the criteria. In our study, the variables Th1/Breg CD24^hi^CD27^+^ and B-memory with switch (dichotomized with a cut-off = 25 cells/µl) maximized the AUC of the 2016 ACR/EULAR CC to 0.953. The value of the 2002 AECG CC was also maximized when considering the variables Bm1 and Tfh (both in percentages), thus increasing the AUC to 0.985.

Although not addressed in this study, the identification of SjS patients in the early phases of the disease could be possible with the characterization of lymphocyte subsets. The identification of a lymphocytic subset distribution profile suggestive of SjS, as we have described, could point to this disease when investigating patients with suspition of SjS.

We acknowledge some limitations in our study that may affect the reproducibility, namely patients’ characteristics such as age, sex, disease duration, current and previous therapies, although our patients were recruited from an outpatient clinic and are representative of the clinical setting. However, the small size of our sample may have limited our ability to obtain more robust results. Also, the use of clinical diagnosis as gold standard may originate an incorporation bias due to the familiarity of the experts with the two specific criteria sets under study.

## Conclusions

Our study confirmed the high degree of agreement between both classification criteria and clinical diagnosis, with 2002 AECG criteria having superior performance than 2016 ACR/EULAR criteria. When specific lymphocyte subpopulations were added to each set of criteria, the mathematical model showed an increase in the criteria performance in discriminating between SjS and Sicca patients . To the best of our knowledge, this is the first study that addresses the effect of lymphocyte subset profiling in the performance of the 2016 ACR EULAR criteria. Although our exploratory study suggests, in a novel approach, the potential use of subsets such as unswitched memory B cells to improve the performance of classification criteria, further studies, preferentially multicentric, are needed to confirm if specific populations could be included as a new weighted item in the ACR/EULAR criteria.

## Materials and methods

### Patient selection

Patients with confirmed or suspected SjS were consecutively selected from a SjS dedicated outpatient clinic. All underwent a multidisciplinary evaluation according to the AECG and ACR/EULAR criteria, including a labial gland biopsy, screening for anti-SSA antibodies, ocular evaluation, and unstimulated salivary flow assessment.

Clinical files were reviewed by the treating rheumatologists (FB, JVP) with patient identity occultation, and by an additional experienced rheumatologist (JCB). A clinical diagnosis was obtained according to at least 2 of the 3 expert physicians and considered the gold standard (GS) in this study. If a clinical diagnosis of SjS was established, the patient was included in the SjS group. Otherwise, the patient was included in the Sicca group.

The exclusion criteria applied to all participants were age under 18 years, hepatitis-C or human immunodeficiency virus infection, pre-existing lymphoma, sarcoidosis, graft-versus-host disease, IgG4-related disease, history of head and neck radiation treatment, current anticholinergic therapy, the use of B-cell-depleting therapies or other biologic disease-modifying anti-rheumatic drugs, and the presence of another systemic autoimmune disease.

Written informed consent was obtained from all participants. The study is in accordance to the Declaration of Helsinki. The study was approved by the Ethics committees of Instituto Português de Reumatologia, Hospital Cuf Descobertas, and NOVA Medical School (no.17/2016/CEFCM).

### Flow cytometry procedures

For the immunophenotyping protocols, peripheral blood samples collected in EDTA-coated tubes were processed and analysed within 24 h of collection. A pre-validated panel of monoclonal antibodies was used for the characterization of T and B-cell subsets, including CD3, CD4, CD8, CD19, CD24, CD27, CD38, CCR6, CCR7, CXCR3, CXCR5, Anti-IgD, and Anti-IgM. A lyse-wash protocol was performed for both T and B-cell characterization. A lyse-no wash single platform strategy with BD Trucount tubes (*BD Biosciences, San Diego CA, USA*) was used to obtain absolute counts of all cell subsets. All samples were acquired in a 4-colour BD FACS-Calibur cytometer (*BD Biosciences*). Whenever appropriate, fluorescence-minus-one control tubes were prepared to assess the positivity of dimer expressions.

CellQuestPro™ (*BD Biosciences*) software was used for acquisition and analysis purposes, and Infinicyt™2.0 (*Cytognos S.L., Salamanca, Spain*) software was also used for more differentiated subset analysis.

Within T-cells (CD3^+^), we characterized CD4^+^ and CD8^+^(CD4^−^) subsets, including Th1 (CXCR3^+^CCR6^−^), Th17 (CXCR3^−^CCR6^+^), Tregs (CD4^+^CD25^hi^CD127^lo^), follicular-like CXCR5^+^CD4^+^ (which we designated Tfh) and CXCR5^+^CD8^+^ (designated Tfc). In the CXCR5^+^ Tfh subset, we also identified Tfh1 (CXCR3^+^CCR6^−^) and Tfh17 (CXCR3^−^CCR6^+^).

B-cells (CD19^+^) were addressed according to the IgD/CD27 classification, where the expression of CD27 and IgD identifies naïve B-cells (CD27^−^IgD^+^), CD27^+^ memory B-cells, unswitched (CD27^+^IgD^+^) and switched memory B-cells, (CD27^+^IgD^−^), and IgD^−^CD27^−^ ("double negative") B-cells^46^. An alternative B-cell classification partially overlaps with the IgD/CD27, and identifies B-cells from Bm1 to Bm5 subsets^47^: Bm1 (IgD^+^CD38^−^), Bm2 (IgD^+^CD38^+^), Bm2′ (IgD^+^CD38^++^), Bm3 + Bm4 (IgD^−^CD38^+^) and finally eBm5 (IgD^−^CD38^+^) and Bm5(IgD^−^CD38^−^).‬ We further characterized, according to the expression of CD24, CD27 and CD38, transitional B cells (CD24^Hi^CD38^Hi^) and Plasmablasts (CD27^+/Hi^CD38 ^Hi^).

The gating strategies are described elsewhere^41^.

### Statistical analysis

An exploratory analysis was carried out for all variables. Continuous variables were described with mean and standard deviation (SD) or median and inter-quantile range (IQR: 25th–75th percentile), as appropriate. Categorical data were presented as frequencies and percentages. Mann–Whitney non-parametric test was used to compare lymphocyte subsets or combinations, between SjS and Sicca patients.

Univariable and multivariable analyses were performed using Firth's bias-reduced logistic regression models, used to deal with the problem of separation inherent to the data. Two univariable models (one for each classification criteria) considered clinical diagnosis (GS) as the dependent variable and each classification criteria as the independent variable. From these univariable models, two multivariable models resulted from adding the lymphocyte subsets or combinations. The discriminative ability, to distinguish between SjS and Sicca patients, of the univariable and multivariable models was compared through the Area Under the Receiver Operating Characteristic Curve (AUC), using DeLong test. A level of significance α = 0.05 was used but, due to the exploratory nature of this study, results with p-values < 0.100 were still considered relevant. Data were analyzed using R software^48^.

### Ethical approval

This study was approved by the Ethics committee of *Hospital CUF Descobertas,* 8/09/2014, Ethics committee of *Instituto Português de Reumatologia,* 3/07/2015 and NOVA Medical School Ethics (nº17/2016/CEFCM).

### Informed consent

All patients have signed an informed consent to participate, according to the Declaration of Helsinki.

## Supplementary Information

Supplementary Information.
