# Supplementary material for: Added value of lymphocyte subpopulations in the classification of Sjögren's syndrome
Source: Sci Rep. 2023 Apr 27;13:6872. doi: 10.1038/s41598-023-31782-7 (PMC10140065; doi:10.1038/s41598-023-31782-7)
Supplement: Supplementary file 1 — Supplementary Information. [file 41598_2023_31782_MOESM1_ESM.docx]

**Supplementary Table 1. Reproduction of the 2002 AECG and 2016 ACR/EULAR criteria**

| **#** | **2002 AECG ^a^** | **2016 ACR/EULAR ^b^** | **Score** |
| --- | --- | --- | --- |
| **1** | Ocular symptoms: a positive response to at least one of the questions:  Have you had daily, persistent, troublesome dry eyes for more than 3 months?  Do you have a recurrent sensation of sand or gravel in the eyes?  Do you use tear substitute more than 3 times a day? |  |  |
| **2** | Oral symptoms: a positive response to at least one of the questions:  Have you had a daily feeling of dry mouth for more than 3 months?  Have you had recurrently and persistently swollen salivary glands as an adult?  Do you frequently drink liquids to aid in swallowing dry food? |  |  |
| **3** | Objective ocular signs – a positive result for at least one of the following two tests:  Schirmer’s I test, performed without anesthesia (≤5 mm in 5 min)  Rose Bengal score or other ocular dye score (≥4 according to van Bijsterveld’s scoring system) | Objective ocular signs:  A positive result for Schirmer’s I test, performed without anesthesia (≤5 mm in 5 min)  Keratoconjunctivitis sicca with:  Ocular staining score ≥3 or van Bijsterveld’s scoring system ≥4 | **1**  **1** |
| **4** | Histopathology: in minor salivary glands (obtained through normal appearing mucosa) focal lymphocytic sialoadenitis, evaluated by an expert histopathologist, with a focus score ≥ 1, defined as number of lymphocytic foci (which are adjacent to normal-appearing mucous acini and contain more than 50 lymphocytes) per 4 mm^2^ of glandular tissue | Labial salivary gland biopsy exhibiting focal lymphocytic sialadenitis with a focus score ≥1 focus/ 4 mm^2^ | **3** |
| **5** | Salivary gland involvement: objective evidence of salivary gland involvement defined by a positive result for at least one of the following diagnostic tests:  Unstimulated whole salivary flow (≤1.5 ml in 15 min)  Parotid sialography showing the presence of diffuse sialectasias (punctuate, cavitary, or destructive pattern), without evidence of obstruction in major ducts  Salivary scintigraphy showing delayed uptake, reduced concentration and /or delayed excretion of tracer | Salivary gland involvement: objective evidence of salivary gland involvement defined by Unstimulated whole salivary flow (≤1.5 ml in 15 min) | **1** |
| **6** | Autoantibodies: presence in the serum of the following antibodies: Antibodies to Ro (SSA) and/or La (SSB) antigens | Autoantibodies: presence in the serum of the following antibodies:  Antibodies to Ro (SSA) | **3** |
|  | ***Classification rules*** | | |
|  | The presence of any 4 of the 6 items is indicative of primary SS, if either item 4 (Histopathology) or 6 (Serology) is positive | The classification of SS, which applies to individuals with signs/symptoms that may be suggestive of SS, will be met in patients who have ≥4 points |  |

**^a^ Adapted from** Vitali C, Bombardieri S, Jonsson R, Moutsopoulos HM, A. *et al.* Classification criteria for Sjögren’s syndrome: a revised version of the European criteria proposed by the American-European Consensus Group. *Ann. Rheum. Dis.* **61**, 554–8 (2002).

**^b^ Adapted from** Shiboski, C. H. *et al.* 2016 American College of Rheumatology/European League Against Rheumatism Classification Criteria for Primary Sjögren’s Syndrome: A Consensus and Data-Driven Methodology Involving Three International Patient Cohorts. *Arthritis Rheumatol.* **69**, 35–45 (2017).

**Supplementary Table 2.** **Comparison of T and B-cell subsets absolute counts in SjS and Sicca groups**

Mann-Whitney nonparametric U test was used for group's comparison. Results are presented as medians and 25^th^ and 75^th^ quartiles. Statistically significant results are indicated in bold.

| **Lymphocyte subsets or combinations of subsets**  Cells/µL, median (25th-75th percentile) | **SjS**  n = 62 | **Sicca**  n = 63 | **Group's comparisons (*p* values)** |
| --- | --- | --- | --- |
|  |  |  | **SjS vs Sicca** |
| **Lymphocytes** | 1679 (1135, 2350) | 2006 (1533, 2277) | **0.089** |
| T-cells | 1192 (768, 1754) | 1429 (1076, 1672) | 0.166 |
| CD4^+^ | 785 (437, 1074) | 889 (708, 11595) | **0.051** |
| CD8^+^ | 431 (319, 632) | 457 (343, 577) | 0.950 |
| B-cells | 187 (113, 264) | 224 (140, 303) | 0.152 |
| **T-cell subsets** |  |  |  |
| Th1 | 260 (161, 418) | 359 (222, 467) | **0.028** |
| Th17 | 125 (87, 224) | 200 (135, 264) | **0.010** |
| Treg | 64 (38, 89) | 69 (47, 84) | 0.436 |
| Tfh (CXCR5^+^CD4^+^) | 135 (83, 205) | 169 (121, 199) | 0.177 |
| Tfh1 | 51 (26, 73) | 55 (34, 67) | 0.600 |
| Tfh17 | 27 (16, 49) | 35 (24, 50) | **0.050** |
| Tfc (CXCR5^+^CD8^+^) | 13 (7, 19) | 12 (9, 17) | 0.894 |
| **B-cell subsets** |  |  |  |
| IgD/CD27 |  |  |  |
| Naive | 122 (63, 195) | 135 (72, 203) | 0.380 |
| Memory | 50 (29, 76) | 66 (43, 97) | **0.021** |
| Unswitched | 23 (12, 38) | 35 (21, 54) | **0.009** |
| Switched | 22 (15, 38) | 30 (20, 47) | **0.045** |
| Bm1-5 |  |  |  |
| Bm1 | 18 (10, 28) | 29 (18, 43) | **<0.001** |
| Bm2 | 109 (59, 172) | 124 (72, 182) | 0.331 |
| Bm2' | 11 (6, 29) | 11 (6, 19) | 0.562 |
| Bm3+Bm4 | 3 (1, 5) | 3 (2,4) | 0.767 |
| eBm5 | 15 (9, 23) | 18 (11, 28) | **0.089** |
| Bm5 | 11 (8, 19) | 15 (10, 22) | **0.079** |
| Bm2+Bm2' | 128 (69, 195) | 139 (81, 198) | 0.479 |
| eBm5+Bm5 | 26 (18, 44) | 34 (23) | **0.071** |
| Bm2+Bm2'/Bm5+eBm5 | 4 (2, 7) | 4 (2, 6) | 0.476 |
| CD24^hi^CD38^hi^ | 8 (4, 18) | 9 (4, 13) | 0.726 |
| CD24^hi^CD27^+^ | 30 (17, 49) | 47 (28, 77) | **0.001** |
| **T-cell/B-cell ratios** |  |  |  |
| T_h_1/CD24^hi^CD27^+^ | 9.75 (5.88, 16.12) | 7.07 (4.39, 10.06) | **0.003** |
| T_h_17/Tregs | 0.26 (0.18, 0.39) | 0.34 (0.22, 0.44) | **0.025** |

Bold numbers represent significance (p-values < 0.05) or relevance (p-values < 0.100); SjS, Sjögren’s syndrome.

**Supplementary Table 3.** **Combination of Immune cell counts with the AECG and ACR/EULAR Classification Criteria**

*Area* *Under the Curve* (AUC) *receiver operating characteristic curve* (ROC) of the comparison of T and B-cell subsets absolute counts in SjS (clinical diagnosis) and Sicca groups (GS vs Sicca). Added value of the addition of each cell subset to the AECG (AUC and p-value) and to the ACR/EULAR criteria (AUC and p-value).

| **Lymphocyte subsets or combinations of subsets**  AUC, 95% CI | **GS vs Sicca** | **Added to 2002 AECG** | | **Added to 2016 ACR/EULAR** | |  |
| --- | --- | --- | --- | --- | --- | --- |
|  | **AUC** | **AUC** | ***p* values** | **AUC** | ***p* values** |  |
|  |  |  |  |  |  |  |
| **Lymphocytes** |  |  |  |  |  |  |
| T-cells | 0.581 (0.477-0.685) | 0.973 (0.938-1) | 0.411 | 0.936(0.887 - 0.984) | 0.361 |  |
| B-cells | 0.590 (0.489-0.690) | 0.964 (0.930-0.999) | 0.630 | 0.917(0.863 - 0.971) | 0.719 |  |
| **T-cell subsets** |  |  |  |  |  |  |
| Th1 | 0.615 (0.515-0.715) | 0.972 (0.938-1) | 0.423 | 0.932 (0.881-0.983) | 0.441 |  |
| Th17 | 0.630 (0.530-0.729) | 0.950 (0.900-1) | 0.967 | 0.930 (0.877-0.982) | 0.479 |  |
| Treg | 0.555 (0.453-0.658) | 0.972 (0.936-1) | 0.446 | 0.938 (0.981-0.894) | 0.325 |  |
| Tfh (CXCR5^+^) | 0.585 (0.482-0.687) | 0.954 (0.904-1) | 0.948 | 0.921 (0.861-0.982) | 0.658 |  |
| Tfh1 | 0.540 (0.437-0.643) | 0.961 (0.919-1) | 0.742 | 0.918 (0.859-0.978 | 0.707 |  |
| Tfh17 | 0.614 (0.512-0.715) | 0.953 (0.902-1) | 0.955 | 0.900 (0.830-0.970) | 0.941 |  |
| Tfc (CXCR5^+^CD8^+^) | 0.502 (0.399-0.605) | 0.968 (0.931-1) | 0.289 | 0.925 (0.873-0.977) | 0.238 |  |
| **B-cell subsets** |  |  |  |  |  |  |
| IgD/CD27 |  |  |  |  |  |  |
| Naive | 0.561 (0.459-0.662) | 0.971 (0.943-0.998) | 0.423 | 0.904 (0.844-0.965) | 0.982 |  |
| Memory | 0.626 (0.526-0.725) | 0.967 (0.930-1) | 0.568 | 0.937 (0.891-0.983) | 0.334 |  |
| Unswitched | 0.637 (0.539-0.735) | 0.969 (0.936-1) | 0.498 | 0.932 (0.884-0.980) | 0.420 |  |
| Switched | 0.614 (0.514-0.714) | 0.954 (0.909-1) | 0.928 | 0.941 (0.897-0.986) | 0.271 |  |
| Bm1-5 |  |  |  |  |  |  |
| Bm1 | 0.675 (0.580-0.770) | 0.964 (0.927-1) | 0.637 | 0.923 (0.871-0.976) | 0.593 |  |
| Bm2 | 0.566 (0.526-0.725) | 0.970 (0.941-0.999) | 0.442 | 0.903 (0.842-0.965) | 0.998 |  |
| Bm2' | 0.515 (0.411-0.618) | 0.962 (0.924-1) | 0.704 | 0.916 (0.860-0.972) | 0.742 |  |
| Bm3+Bm4 | 0.471 (0.368-0.573) | 0.970 (0.937-1) | 0.476 | 0.940(0.895-0.985) | 0.289 |  |
| eBm5 | 0.599 (0.499-0.700) | 0.961 (0.922-1) | 0.728 | 0.934 (0.886-0.981) | 0.395 |  |
| Bm5 | 0.594 (0.493-0.696) | 0.956 (0.910-1) | 0.890 | 0.397 (0.890-0.983) | 0.344 |  |
| Bm2+Bm2' | 0.552 (0.450-0.654) | 0.970 (0.941-0.999) | 0.452 | 0.904 (0.844-0.965) | 0.980 |  |
| eBm5+Bm5 | 0.602 (0.501-0.703) | 0.958 (0.916-1) | 0.821 | 0.936 (0.890-0.982) | 0.352 |  |
| CD24^hi^CD38^hi^ | 0.497 (0.392-0.601) | 0.961 (0.921-1) | 0.744 | 0.925 (0.871-0.979) | 0.567 |  |
| CD24^hi^CD27^+^ | 0.668 (0.572-0.764) | 0.967 (0.932-1) | 0.560 | 0.932 (0.885-0.980) | 0.410 |  |
| **T-cell/B-cell ratios** |  |  |  |  |  |  |
| T_h_1/CD24^hi^CD27^+^ | 0.638 (0.539-0.736) | 0.960 (0.923-0.997) | 0.741 | 0.895 (0.832-0.958) | 0.837 |  |
| T_h_17/Tregs | 0.609 (0.509-0.709) | 0.967 (0.932-1) | 0.562 | 0.937 (0.889-0.984) | 0.347 |  |

GS, Gold-Standard; AUC, area under the Receiver-Operating-Characteristic Curve; IC: Interval of Confidence; GS, Gold-Standard; AUC, area under the Receiver-Operating-Characteristic Curve; IC: Interval of Confidence, AECG, American-European Consensus Group classification.
